# Association between underlying dentin shadows (ICDAS 4) and OHRQoL among adolescents from southern Brazil

**DOI:** 10.1590/1807-3107bor-2024.vol38.0046

**Published:** 2024-06-24

**Authors:** Patrícia Kolling MARQUEZAN, Letícia Donato COMIM, Débora Nunes de Oliveira RACKI, Ângela DALLA NORA, Luana Severo ALVES, Julio Eduardo do Amaral ZENKNER

**Affiliations:** (a) Universidade Federal de Santa Maria – UFSM, Department of Microbiology and Parasitology, Santa Maria, RS, Brazil; (b) Universidade Federal de Santa Maria – UFSM, Department of Restorative Dentistry, Santa Maria, RS, Brazil; (c) Universidade Federal de Santa Maria – UFSM, Department of Stomatology, Santa Maria, RS, Brazil

**Keywords:** Dental Caries, Adolescent, Quality of Life, Cross-Sectional Study, Epidemiology

## Abstract

This study aimed to assess the association between underlying dentin shadows (UDS) and oral health-related quality of life (OHRQoL) among 15-19-year-old adolescents from southern Brazil. This population-based cross-sectional study included a representative sample of 1,197 15–19-year-old adolescents attending 31 public and private schools from Santa Maria, Brazil. The Oral Health Impact Profile-14 (OHIP-14) was used to evaluate the OHRQoL, and clinical examinations were performed by two calibrated examiners (intra/interexaminer kappa values for caries examination ≥ 0.80) to diagnose UDS (ICDAS code 4 caries lesions). Sociodemographic information and clinical characteristics (overall caries experience, traumatic dental injury, malocclusion, and gingivitis) were also collected as adjusting variables. Multilevel Poisson regression models were used to assess the association between UDS and OHRQoL. Rate ratios (RR) and 95% confidence intervals (CI) were estimated. The UDS prevalence was 8.8% (n = 106 adolescents). In the adjusted models, adolescents with UDS had poorer OHRQoL than those without UDS, and the strength of the association was dependent on the number of lesions per individual. Individuals with 1-2 UDS had a mean OHIP-14 score 8% higher (RR = 1.08; 95%CI: 1.01–1.17) than adolescents without UDS, while those with 3-4 UDS had a mean score 35% higher (RR = 1.35; 95%CI: 1.12–1.63). This negative association was related to physical disability, psychological disability, social disability, and handicap domains. This study showed that UDS was associated negatively with OHRQoL among 15–19-year-old adolescents from southern Brazil. The negative effect of UDS on OHRQoL emphasizes the importance of addressing issues regarding OHRQoL even in the posterior teeth of adolescents.

## Introduction

The concept of health includes biopsychosocial models in which physical, emotional, and social well-being is interconnected.^1^ Oral health strongly influences this process, given its bearing on the ability to speak, eat, and socialize.^2^ Poor oral health directly affects one’s oral health-related quality of life (OHRQoL), defined as a multidimensional construct that describes an individual’s subjective perspective, based on his/her oral symptoms and experiences.^3^ Major challenges during adolescence involve achieving good oral health and improving the OHRQoL, since this stage of life represents a period of constant changes, adaptations to new environmental and psychological structures, and the construction of the adolescents’ identity.^4^ Poor oral health behaviors in this age group make adolescence a high-risk period for the development of caries lesions.^5^

An estimated 2.3 billion people worldwide suffer from tooth decay in the permanent dentition.^6^ According to Brazil’s last national oral health survey, 35.8% of adolescents aged 15–19 years had decayed teeth.^7^ Epidemiological studies have consistently found that caries is negatively associated with OHRQoL in adolescents.^8-15^ The degree of impact of caries on OHRQoL is directly related to the number of affected teeth,^16,17^ lesion severity,^18^ intraoral distribution^19^ and dental pain.^20^ Not only cavitated caries lesions, but also moderate caries lesions, such as underlying dentin shadows (UDS), can have a potentially negative impact on OHRQoL. Classified as code 4 by the International Caries Detection and Assessment System (ICDAS),^21^ UDS appear as a discolored dentin shadow visible through an apparently intact enamel surface, which may or may not show signs of localized breakdown. The darkened area is an intrinsic shadow that may appear gray, blue or brown, and may influence the self-perception of oral health, mainly among adolescents. Despite this assumption, no previous study has investigated this issue to date.

Although the prevalence of this lesion was recently found to be low in young populations,^22,23^ its possible relationship with OHRQoL must be investigated. Therefore, the aim of this study was to assess the association between UDS in the occlusal surfaces of permanent posterior teeth and OHRQoL among 15–19-year-old adolescents from southern Brazil. The hypothesis was that adolescents who present UDS have poorer OHRQoL.

## Methods

### Study design and sample

A population-based cross-sectional study was carried out to assess the oral health status of adolescents aged 15–19 years old from Santa Maria, a mid-sized city located in southern Brazil. All the 37 high schools in the municipality were invited to participate in the study (26 public and 11 private), 31 of which agreed to participate (22 public and 9 private).

A total of 1,066 adolescents were found to be needed for the study. The sample size calculation used the following parameters: a prevalence rate of 50% (worst case scenario), a 95% confidence interval (CI), a power of 80%, and a precision level of 3%. Considering a non-participation rate of 50%, 1,600 adolescents were invited to participate.

### Eligibility criteria

Adolescents born in the years 1999–2003, attending any school period (morning, afternoon, or night), and not using fixed orthodontic appliances were considered eligible. Students with special needs (cognitive or physical impairments that prevented them from answering the questionnaire, or from being clinically examined in the school setting) were not considered eligible for the study. A list of all eligible schoolchildren was compiled for each school, and those eligible were selected using a table of random numbers (http://www.random.org).

### Data collection

Data collection was conducted from March to November 2018, and included questionnaires and a clinical examination. A self-administered questionnaire was used to gather information on sociodemographic characteristics (sex, age, skin color, mother’s level of education, and socioeconomic status). It was sent to the parents/legal guardians of the selected students to be completed at home.

The Oral Health Impact Profile-14 (OHIP-14) was used to evaluate the OHRQoL. This questionnaire is an instrument that measures people’s perception of the social impact of oral disorders on their well-being.^24^ The OHIP-14 was translated into Brazilian Portuguese and validated for the language.^25^ It is the short version of a longer instrument, and is composed of 14 questions related to seven conceptually formulated dimensions: functional limitation, physical pain, psychological discomfort, physical disability, psychological disability, social disability, and handicap. The answers to each question are based on a Likert scale grade: never = 0 point, rarely = 1 point, sometimes = 2 points, often = 3 points, and always = 4 points. The sum of the answers provides a score ranging from 0 to 56 points – the higher the score, the poorer the OHRQoL.^24-26^ The OHIP-14 was applied to the selected adolescents in the school setting, just before the clinical oral examination.

Clinical examinations were conducted at the schools, with the students in a supine position, using portable equipment (artifical light and air compressor). A sterile clinical mirror and a periodontal probe were also used. The teeth were cleaned with a toothbrush and dried prior to caries examination. Cotton rolls were used to ensure proper moisture control, and dental caries was recorded by two calibrated examiners. Caries examination included the recording of both non-cavitated and cavitated lesions, as well as caries activity assessment.^27^ In addition, the presence of UDS was also recorded, as defined by the ICDAS.^21^ In addition, traumatic dental injuries (TDI) were assessed by using the O’Brien classification,^28^ gingivitis, by using the gingival bleeding index (GBI),^29^ and malocclusion, according to the dental aesthetic index (DAI).^30^

### Training and calibration

Clinical examination was performed by two calibrated examiners (DNOR, ADN). Training sessions using photographs, study models, and clinical exams were performed under the supervision of a benchmark examiner. The examiners’ calibration was assessed in 10 adolescents before the start of the study, and its continued validity during the survey was checked by repeated examinations of 20 schoolchildren out of every 400 examined (totaling 5% of the sample). The minimal time interval between examinations was 7 days. The minimal value of the intraexaminer kappa coefficient was 0.81 for dental caries and 0.89 for TDI, while the minimal interexaminer kappa value was 0.80 for dental caries and 0.77 for TDI. The minimal intraclass correlation coefficient for DAI measures was 0.89 (intraexaminer) and 0.87 (interexaminer). As for the GBI, training was performed under the supervision of an experienced periodontist, but no calibration was performed due to the temporary nature of the condition.

### Ethical aspects

The Research Ethics Committee of the Federal University of Santa Maria approved the study protocol (# 2.178.299). All participants (≥ 18 years old) or their parents/legal guardians signed a written informed consent form. Underage participants signed a written assent form. The students received a report of their oral health status, and were referred to dental treatment when needed.

### Data analysis

The outcome of this study was OHRQoL, measured as the overall and domain-specific OHIP-14 scores. The main predictor variable was the extent of UDS, defined as the number of permanent posterior teeth with occlusal UDS per individual (0, 1–2 or 3–4).

Sociodemographic adjusting variables included sex (male or female), age (15, 16, 17 or 18–19 years), skin color (non-white or white), and socioeconomic status (SES). The SES categories were defined by using the cutoff points proposed by the standard Brazilian economic classification,^31^ and households were classified as having a low (≤ 16), mid-low (≥ 17 to ≤ 22), mid-high (≥ 23 to ≤ 28) or high (≥ 29 point) SES. Clinical adjusting variables were malocclusion (absent [DAI ≤ 25], or present [DAI > 25]),^32^ dental caries experience at the cavity level (absent [DMFT = 0], or present [DMFT ≥ 1]),^30^ and gingivitis (absent [<10% of sites with bleeding on probing], or present [≥ 10% of sites with bleeding on probing]).^33^ Figure presents all the variables included in the study.

**Figure:**
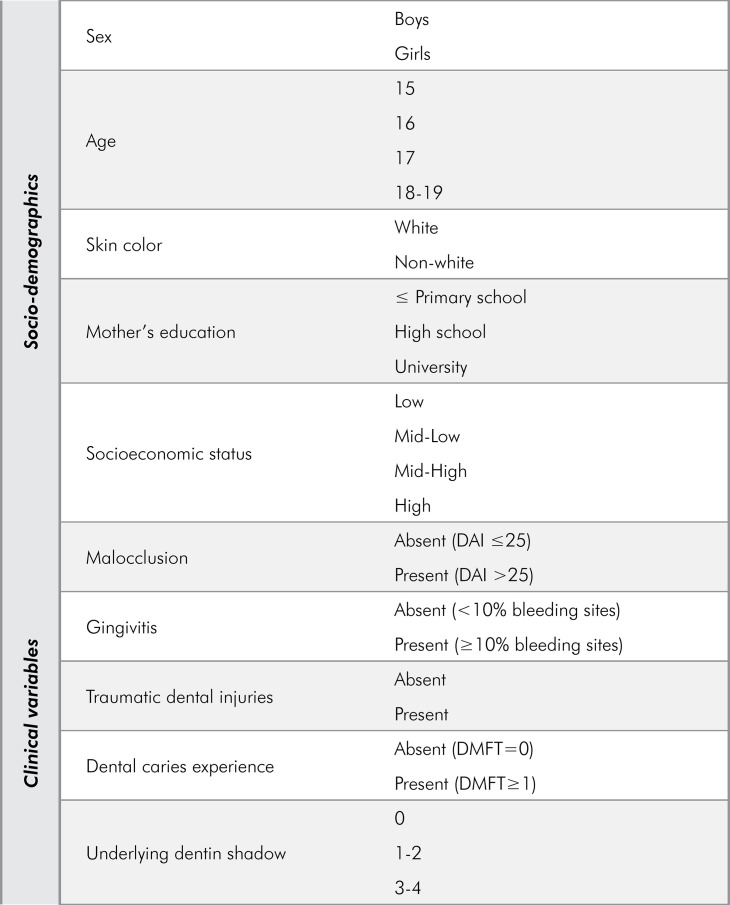
Variables included in the study (DAI, Dental Aesthetic Index; DMFT, Decayed, Missing or Filled Teeth Index).

Data analysis was performed using STATA software (Stata 11.1 for Windows; Stata Corporation, College Station, USA), which used survey commands that performed all the descriptive analyses according to the survey design. A weight variable was used to adjust for potential bias in the population estimates, based on the probability of selection and population distribution according to sex and school type. The overall and the domain-specific OHIP-14 mean scores and standard errors (SE) were reported. Preliminary analysis comparing the mean OHIP-14 scores among the categories of the predictors was done using the Wald test.

The association between UDS (main predictor variable) and OHRQoL was assessed using multilevel Poisson regression models. The multilevel model considered the adolescent as the first-level unit, and the school as the second-level unit. The multilevel model used the scheme of fixed effect with random intercept. Unadjusted and adjusted rate ratios (RR) and 95% confidence intervals (CI) were estimated. All the variables were included and maintained in the adjusted model, irrespective of their p-values. The level of significance was set at 5%.

## Results

A representative sample of 1,197 out of the 1,656 15–19-year-old adolescents was included in the study, thus representing a response rate of 72.3%. A total of 106 adolescents presented at least one occlusal UDS in a permanent posterior tooth, corresponding to 8.8% (95%CI: 0.65–0.13) of the sample. The mean OHIP–14 score was 8.25 (95%CI: 7.75–8.75), ranging from 0 to 49. Table 1 summarizes the distribution of the sample and the OHIP–14 scores, according to sociodemographics and clinical characteristics. OHIP–14 scores differed significantly among the categories of all the variables studied, except for gingivitis, TDI, and UDS.

**Table 1:** Sample distribution and OHIP–14 scores by predictor variables.

Variables	n (%)	Mean (SE)**	Range
Socio–demographics			
Sex			
Boys	513 (42.9)	7.36 (0.40)^a^	0–38
Girls	684 (57.1)	9.10 (0.32)^b^	0–49
Age			
15	276 (23.2)	7.67 (0.56)^abc^	0–36
16	379 (31.7)	7.51 (0.31)^b^	0–49
17	367 (30.7)	8.58 (0.38)^cd^	0–43
18–19	175 (14.6)	10.2 (0.76)^d^	0–40
Skin color*			
White	384 (33.0)	9.01 (0.40)^a^	0–49
Non–white	779 (67.0)	7.90 (0.26)^b^	0–43
Mother’s education*			
≤ Primary school	577 (50.2)	8.74 (0.35)^a^	0–49
High school	380 (33.1)	8.20 (0.36)^a^	0–34
University	192 (16.8)	6.53 (0.54)^b^	0–33
Socioeconomic status*			
Low	201 (17.4)	10.35 (0.53)^a^	0–40
Mid–Low	320 (27.6)	8.70 (0.45)^b^	0–43
Mid–High	302 (26.1)	8.26 (0.24)^b^	0–38
High	335 (29.0)	6.88 (0.47)^c^	0–49
Clinical variables			
Malocclusion			
Absent (DAI ≤ 25)	293 (24.5)	7.01 (2.50)^a^	0–32
Present (DAI > 25)	904 (75.5)	8.66 (0.33)^b^	0–49
Gingivitis			
Absent (<10% bleeding sites)	1,031 (86.1)	8.07 (0.27)^a^	0–49
Present (≥10% bleeding sites)	166 (13.9)	9.35 (0.70)^a^	0–37
Traumatic dental injuries			
Absent	993 (83.0)	8.07 (0.30)^a^	0–49
Present	204 (17.0)	9.10 (0.54)^a^	0–43
Dental caries experience			
Absent (DMFT = 0)	641 (53.5)	6.81 (0.32)^a^	0–43
Present (DMFT ≥ 1)	556 (46.4)	9.95 (0.39)^b^	0–49
Underlying dentin shadow			
0	1,091 (91.1)	8.20 (0.24)^a^	0–49
1–2	95 (8.0)	9.00 (1.20)^a^	0–43
3–4	11 (0.9)	10.90 (3.18)^a^	0–36
Total	1,197 (100)	8.24 (0,25)	0–49

SE: standard error; DAI: Dental aesthetic index; DMFT: decayed, missing, and filled teeth index. *Missing data. **Taking into account the sampling weight. Different letters indicate statistically significant difference between categories (p < 0.05, adjusted Wald test).

The association between UDS and the overall and domain–specific OHIP–14 scores is shown in Table 2. In the unadjusted models, the presence of 1–2 UDS lesions was significantly associated with social disability, while the presence of 3–4 UDS lesions was significantly associated with psychological disability and handicap. The adjusted models, including sociodemographic and clinical variables, showed that adolescents with 1–2 UDS had a poorer OHRQoL than adolescents without UDS in the social disability domain (RR = 1.30; 95%CI: 1.02–1.64). In comparison, adolescents with 3–4 UDS lesions had a poorer OHRQoL for the physical disability (RR = 1.74; 95%CI: 1.04–2.91), psychological disability (RR = 1.72; 95%CI: 1.13–2.64), social disability (RR =1 .82, 95%CI: 1.00–3.30) and handicap domains (RR = 2.32, 95%CI: 1.24–4.34). Overall, adolescents with 1–2 UDS had an OHIP–14 score 8% higher than individuals without UDS (RR = 1.08; 95%CI: 1.01–1.17), and those with 3–4 UDS presented a more notable difference, with 35% higher mean scores than adolescents without UDS (RR = 1.35; 95%CI: 1.12–1.63). As for the other variables included in the adjusted models, all of them were significantly associated with the overall OHIP–14 score (p < 0.05).

**Table 2:** Association between UDS and both domain–specific and overall OHIP–14 scores among Brazilian adolescents (multilevel Poisson regression analysis).

Variables	Unadjusted			Adjusted^†^		

	RR	95%CI	p-value	RR	95%CI	p-value
Functional limitation						
0 UDS	1.00				1.00	
1–2 UDS	1.16	0.91–1.48	0.24	1.12	0.87–1.44	0.39
3–4 UDS	1.12	0.55–2.28	0.75	1.22	0.60–2.49	0.58
Physical pain						
0 UDS	1.00				1.00	
1–2 UDS	1.15	1.00–1.32	0.05	1.12	0.97–1.29	0.12
3–4 UDS	1.15	0.78–1.70	0.47	1.22	0.82–1.82	0.32
Psychological discomfort						
0 UDS	1.00				1.00	
1–2 UDS	1.13	0.99–1.28	0.07	1.07	0.94–1.23	0.30
3–4 UDS	0.91	0.61–1.37	0.66	0.94	0.62–1.42	0.76
Physical disability						
0 UDS	1.00				1.00	
1–2 UDS	0.87	0.68–1.12	0.28	0.84	0.66–1.09	0.19
3–4 UDS	1.64	1.00–2.72	0.05	1.74	1.04–2.91	0.03
Psychological disability						
0 UDS	1.00				1.00	
1–2 UDS	1.10	0.92–1.31	0.29	1.06	0.88–1.27	0.54
3–4 UDS	1.58	1.04–2.41	0.03	1.72	1.13–2.64	0.01
Social disability						
0 UDS	1.00				1.00	
1–2 UDS	1.36	1.08–1.71	0.01	1.30	1.02–1.64	0.03
3–4 UDS	1.76	0.98–3.16	0.06	1.82	1.00–3.30	0.047
Handicap						
0 UDS	1.00				1.00	
1–2 UDS	1.25	0.93–1.66	0.14	1.19	0.88–1.61	0.25
3–4 UDS	2.06	1.12–3.82	0.02	2.32	1.24–4.34	0.008
OHIP-14						
0 UDS	1.00				1.00	
1–2 UDS	1.12	1.122–1.125	< 0.001	1.08	1.01–1.17	0.03
3–4 UDS	1.28	1.10–1.54	0.008	1.35	1.12–1.63	0.002

RR: Rate ratio; CI: Confidence interval; UDS: Underling dentin shadow. †Estimates are adjusted for sex, age, skin color, socioeconomic status, dental caries experience, traumatic dental injuries, malocclusion, and gingivitis. Bold numbers identify p–values < 0.05.

## Discussion

This study assessed the association between UDS and OHRQoL among 15–19–year–old adolescents from southern Brazil. Our main finding was that individuals with UDS had poorer OHRQoL than those without UDS, even after adjusting for important cofactors, thus confirming the study hypothesis. To the best of our knowledge, this was the first study to assess this association.

Quality of life indicators related to oral health are fundamental for understanding and measuring the physical and psychological influence of oral diseases in aggravating individual lives, particularly, joy of living, possibility of speaking, chewing capacity, social inclusion,^20^ and, more recently, happiness.^35^ In the present study, we found that adolescents with UDS had higher OHIP–14 scores than those without UDS, and that the magnitude of the association was related to the number of affected teeth – the higher the number of UDS, the greater the negative association with OHRQoL. After adjusting for important sociodemographic factors and oral conditions, we found that adolescents with 1–2 UDS had 8% higher overall OHIP–14 scores, while those with 3–4 UDS had 35% higher scores. Since it is not uncommon for many adolescents to feel embarrassed to admit issues regarding their appearance,^36^ this is a plausible finding. The clinical aspect of these lesions, in black, blue or gray shadows, may make adolescents feel frustrated or worried about their teeth. This concern regarding aesthetics/appearance may explain the association between UDS and both the psychological disability and social disability domains, even considering that molars are the most commonly affected teeth.^23^ Adolescents with 3–4 UDS had approximately 72% higher OHIP–14 scores in the psychological disability domain than their counterparts without UDS, thus indicating that they were more likely to have difficulty relaxing, or to feel embarrassed because of tooth–related problems. As for the social disability domain, a significant gradient was observed, namely that adolescents with 1–2 UDS and those with 3–4 UDS presented 30% and 82% higher scores, respectively, than those without UDS. This means that these individuals with UDS, versus those without it, were more commonly irritated by other people, or had more difficulty performing their usual activities because of teeth–related problems. It can be speculated that even the association with the handicap domain might be related to aesthetic issues, since it involves a feeling of being less satisfied with life due to oral problems.

Although we are dealing with UDS in posterior teeth, aesthetic–related demands for restorative procedures in posterior teeth are routine in clinical practice. This clearly indicates that aesthetics in the posterior segment is a concern for some individuals. Replacement of amalgam by tooth–colored restorations for aesthetic reasons have been reported in the literature.^37,38^ Similarly, Spelid et al. showed that aesthetics were important to young Norwegian and Danish patients, even in scenarios dealing with restorations in posterior teeth.^39^ Their study was designed to examine how dental professionals and young patients valued three attributes of dental restorations, namely expected longevity, appearance, and risk of adverse reaction. The authors showed that young patients were willing to sacrifice longevity much more than dentists, if it meant avoiding a highly visible restoration.^39^ The increasing number of studies dealing with the aesthetic properties of composite resin restorations in molars is further evidence that aesthetics matters even in posterior teeth.^40^ In this sense, dental professionals should be aware that patients may seek treatment for UDS in posterior teeth because of aesthetics/appearance.

As previously suggested in the literature, most UDS may present either no radiolucency, or radiolucency at the enamel–dentin junction, with only a few cases showing an obvious spread to dentin.^43,44^ Unfortunately, radiographs of the sample cannot be obtained because of the field conditions under which epidemiological studies are conducted. However, considering the lack of an association between the OHIP–14 scores and both the functional limitation and physical pain domains, it is likely that the UDS observed in this study were not deep caries lesions, as corroborated by the literature on this topic.^43,44^ Considering that most UDS present no radiographically evident spread to dentin,^43,44^ and that the progression rate is low, as recently shown by our research group,^45^ the indication of operatory treatment should be avoided whenever possible to avert the repetitive restorative cycle, mainly among young patients. Sharing this knowledge with patients could make them less concerned about their oral health, and ultimately improve their quality of life.

Our study was composed of a representative sample of 1,197 adolescents attending public and private schools at an undetermined school period, unlike other studies, which included only public school attendees.^20^ In addition, although previous studies showed the relationship between dental caries and OHRQoL,^8^ none specifically assessed UDS. Furthermore, we carried out a clinical examination protocol that included dental cleaning and drying, highly reproducible examiners, and a validated questionnaire to assess OHRQoL, thus providing methodological consistency and high internal validity. Another strength of this study was the statistical adjustment for a set of other variables that admittedly could explain the OHIP–14 scores. The lack of radiographic examination is a limitation of this study, as previously discussed. Knowledge of the radiographic presence/depth of the clinically detected UDS would help better understand the association between UDS and OHRQoL found in this study. It should also be borne in mind that this was a cross–sectional study, and that no causal relationship can be established. In conclusion, the present study showed that adolescents with UDS had poorer OHRQoL than those without UDS – the higher the number of lesions, the stronger the association.
